# Arousal and Subjective Significance Shapes Stimuli Interpretation across Warmth Vs. Competence Dimensions

**DOI:** 10.1007/s12144-016-9553-9

**Published:** 2017-01-14

**Authors:** Kamil K. Imbir

**Affiliations:** Faculty of Psychology, University of Warsaw, 5/7 Stawki St, 00-183 Warsaw, Poland

**Keywords:** Social cognition, Ambiguous task processing, Priming effect, Duality of mind

## Abstract

The current study sought to examine the impact of incidental activation defined as arousal and subjective significance, both represented in connotations of verbal materials, on social perception of unknown and thus ambiguous objects, in terms of two basic dimensions of social cognition: warmth and competence. Arousal was expected to promote interpretation of ambiguous stimuli in terms of warmth, while subjective significance in terms of competence. This means that both activation and social perception may be treated as examples dual-mind systems functioning. Sixty participants (30 women) were exposed to two subsequent tasks. The first involved memorizing 135 words (manipulation prepared in a factorial design contrasting 3 levels of arousal and 3 levels of subjective significance), and the second involved guessing the meaning of hexagram stimuli derived from Far East culture. An increasing level of arousal caused participants to interpret stimuli as increasingly related to warmth, while an increasing level of subjective significance led to interpretations more related to competence. This study shows that two distinct types of activation may trigger different interpretations of social stimuli, which means that there is a link between both types of processes. This finding is of great importance for the dual-mind approach, showing that a multitude of dualities indentified thus far may be related one another. Therefore, it is justified to treat them as emanations of more general mental systems.

**Electronic supplementary material:**

The online version of this article (doi:10.1007/s12144-016-9553-9) contains supplementary material, which is available to authorized users.

## Introduction

When thinking about the mind, the crucial issue is to understand the mechanisms of its functioning, because this may provide a chance to predict the consequences of certain events, including social ones. This paper aims to search for the consequences of incidental activation for the perception of other people, in terms of two fundamental dimensions of warmth and competence (Fiske et al. 2007; Wojciszke 2005). The theoretical foundation for this study was the dual-processes theory framework (e.g. Epstein 2003; Kahneman 2011) accounting for a huge variety of social processes (Gawronski and Creighton 2013).

## Dual Systems of Mind and Their Activation Mechanisms

Among dual-processes theories, distinguishing between automatic vs. controlled processing modes (e.g. Bargh et al. 1996; Gawronski and Creighton 2013; Kahneman 2011; Strack and Deutsch 2004), there are domain specific models of functioning, like decision-making (e.g., Slovic et al. 2002), reasoning (e.g., Darlow and Sloman 2010) or judging (Lieberman 2003), as well as more general views on how the mind works in different aspects of functioning (c.f. Epstein 2003; Kahneman 2003, 2011). For example, Epstein (2003) postulated two broad personality systems: experiential and rational minds, each with different characteristics related to respectively automated and controlled processing modes (Gawronski and Creighton 2013). The experiential mind may work even without language and is based on currently experienced feelings (Epstein 2003; Kahneman 2003). Such processing is automatic in nature (Bargh 1994; Gawronski and Creighton 2013; Moors and De Houwer 2006), based on associations (Strack and Deutsch 2014), and is, thus, fast and prioritized (Kahneman 2003). It appears without effort (Kahneman 2011) and dominates everyday experience (Epstein 2003). The experiential mind is responsible for giving a quick and approximated response to the task an individual is facing (Kahneman 2011; Lieberman 2003). The rational mind is language centered (Epstein 2003), based on propositional mechanisms (Strack and Deutsch 2014), and strictly follows the rules of formal logic (Darlow and Sloman 2010; Epstein 2003; Kahneman 2011). Such processing is controlled in nature (in other words systematic or slow: Kahneman 2003), and is, thus, less salient, because it consumes energy through effortful operations required to achieve algorithmic answers to the problem (Kahneman 2011). Answers given by a rational mind are, in most cases, accurate and precise (Slovic et al. 2002; Strack and Deutsch 2004), thus, these responses or choices are best at any given time, but their creation costs a lot of energy (Kahneman 2003). For that reason, systematic processing of rational mind almost always appear after the simpler, automatic processing guided by the experiential mind (Epstein 2003; Kahneman 2011; Lieberman 2003), especially when the former fail to give a satisfactory answer or only when a situation is important enough to bother ourselves.

Considering the role of experiential and rational minds in information processing, we have to discuss the role of activation accompanying both minds (Imbir 2016a). The role of activation is to provide energy that enhances processing specific to the requirements of the mental system engaged. In the literature, there is a debate about the nature of activating mechanisms, especially those guiding automatic and effortless processing of experiential mind in comparison to those involved with the controlled and effortful processing of rational mind (c.f. Imbir 2015a, 2016a). Arousal is postulated to be the type of activation (c.f. Epstein 2003; Osgood et al. 1957) specific to the experiential mind (Epstein 2003; Imbir 2016a). Arousal is defined as a level of energy associated with certain objects, providing the opportunity to activate simple processes (often physiological) in order to face the threat to life or to appeal to a potential sexual partner. Such activation does not need language to occur and often disrupts higher cognitive processes like attentional control (Jefferies et al. 2008) or cognitive control in a modified Stroop Task (Imbir 2016b). Epstein (2003), in his experiential vs. rational mind model, claimed that arousal is the factor that shifts the balance towards the experiential mind. In other words, facing arousing objects, the experiential, effortless processing is activated and drives our responses to stimulation. The source of activation of the effortful and controlled processing of the rational mind remains an open question (Imbir 2016a), concerning the reasons for engaging in such complex, and thus demanding, forms of processing (Kahneman 2011). An attempt to solve this problem was the introduction of the subjective significance concept (Imbir 2015a, 2016a) as a manifestation of rational mind activation that can be easily measured with different types of stimuli using the Self-Assessment Manikin (SAM) scale (c.f. Imbir 2015a). In this approach, subjective significance is defined as the “attitude toward an object that renders it important and significant, thereby, meriting the investment of energy in accurate systematic processing” (Imbir 2016b, p. 2). Simply, rational mind activation and the type of enhanced processing have to be based on congruent mechanisms. Rational mind processing is based on language, verbalization (Epstein 2003), and propositional mechanisms (Strack and Deutsch 2014), thus, rational mind activation needs to be based on language related mechanisms, including appraising the importance of the situation (Imbir 2016a, 2016b). Subjective significance was found to be a property of stimuli that is independent from arousal, in terms of low correlations between assessments of these two dimensions made by participants in affective norms studies (Imbir 2016c).

## Warmth and Competence in Social Perception: Model of Relations between Incidental Activation and Ambiguous Stimuli Interpretation

Social psychology has identified that our social cognition and social perception can mostly be accounted for by two dimensions concerning how we judge other people: warmth and competence (Fiske et al. 2007; Wojciszke 2005). Warmth represents socially related traits connected with wellbeing in group functioning and personal contacts, while competence represents ability related traits and attributes of people from our social environment connected with effectiveness of task realization. Studies found that both dimensions explains almost all the variance in favorability ratings (Wojciszke 2005), and a majority of variance in social perception, including approach-–avoidance tendencies (Cacioppo et al. 1997; Peeters 2002) or inferences about the motives of other people (Reeder et al. 2002). It seems that warmth is a more prioritized dimension when perceiving others (Fiske et al. 2007) because, from an evolutionary point of view, it is more important to get to know other people’s intentions (bad or good) than other people’s abilities to realize those intentions. Subjects have quicker reaction times for warmth related words compared to competence related ones, when they were discriminated from pseudo-words in the lexical decisions paradigm (Ybarra et al. 2001). Moreover, very brief (100 ms) exposition of faces allows one to formulate more reliable assessments of trustworthiness (a trait linked with warmth) compared to competence (Willis and Todorov 2006).

**Fig. 1:**
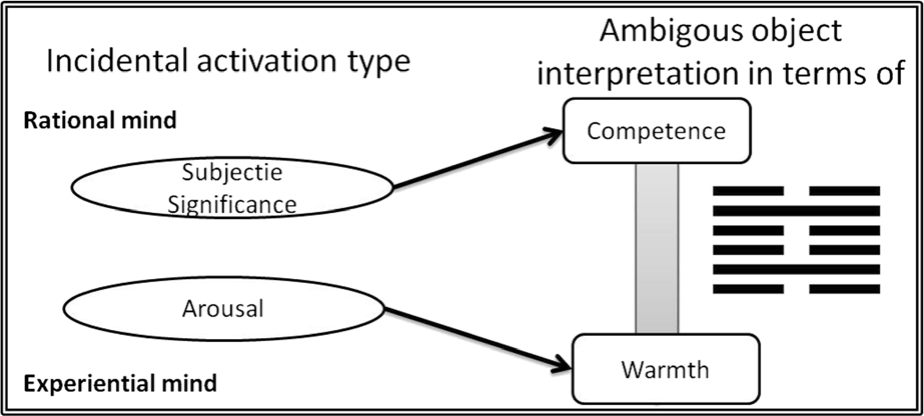
Theoretical model of relation between activation mechanisms of experiential and rational minds and interpretation of ambiguous stimuli in terms of Warmth vs. Competence

This prioritization of warmth judgments over competence assessments suggests that perception of the warmth of others may be based on experiential mind processing mechanisms, while competence perception of others may be related to rational mind processing (c.f. Epstein 2003; Kahneman 2011). To check this intuition, the activational aspect of these two types of process were taken into consideration, because the activation specific to each mental system should trigger a specific mechanism of processing (Epstein 2003), and, thus, the perception in terms of system specific dimension (c.f. Antosz and Imbir 2016). Figure 1 present the theoretical predictions concerning the relations between activation and social perception.

The important issue is how to search for activation outcomes for social perception, since most of known stimuli from our social environment is already interpreted in the context of warmth and competence. This question has been addressed here using two different methodologies: incidental affect elicitation (c.f. Ferguson et al. 2005) and processing of ambiguous objects (Antosz and Imbir 2016; Błaszczak and Imbir 2012). Incidental affect is a term for affective reaction caused by stimulation unrelated to the current task. Such an approach allows for manipulating the affective state of individuals even without the participants’ conscious recognition of changes made, thus, measuring the incidental outcomes of affective reactions occurring in everyday situations. Merely viewing the stimuli (Russell 2003) should influence the actual affective state of individuals, in both valence and activation dimensions (c.f. Ferguson et al. 2005; Imbir 2015b, 2016b). Incidental affect outcomes are also broadly distributed, including processes from food intake (Garg et al. 2007), through categorizations (Kenworthy et al. 2003) and information acquisition (Lee and Sternthal 1999), to social perception (Ferguson et al. 2005).

A good testing ground for associations between social perception and duality of activation may be specific types of tasks involving ambiguity (Pauker et al. 2010), originally introduced by Murphy and Zajonc (1993); see also Błaszczak and Imbir 2012; Payne et al. 2005). When asking participants about the meaning of stimuli unknown to them, such as graphical signs derived from distant cultures (e.g. Chinese ideograms or hexagrams), we may expect that the reaction will be biased by the type of process primed. After suboptimal presentation of faces expressing negative or positive emotions, participants rated ideograms as referring to worse or better objects respectively (Murphy and Zajonc 1993). Other studies showed a tendency to relate hexagrams more to the self when primed by positive faces and less to the self when primed by negative faces (Błaszczak and Imbir 2012). Taking the above into consideration, one may expect that ambiguous stimuli, such as hexagrams (c.f. method section) may be interpreted in different ways, due to priming stimuli properties.

## Aim and Hypothesis

The aim of the current study was to test predictions concerning activation mechanisms of experiential and rational mind systems, and their role in the interpretation of stimuli thought to represent other people’s traits in terms of their warmth and competence. To do so, ambiguous hexagram sign processing was applied with superficially unrelated presentation of activation charged words. Arousal related words were expected to promote perception in terms of warmth, due to eliciting experiential mind mechanisms. Subjective significance was expected to promote perception in terms of competence, due to eliciting rational mind mechanisms. The link between activation types and mind types was assumed to be crucial in ambiguity interpretation (Imbir 2016a). No specific expectations were formulated for reaction latencies. The importance of the study presented here is based on the approach focused on searching for links between different dualities described in the literature (of activation mechanisms or perceptions of others), indicating that all of them are derivates of two more general mind systems (experiential and rational: Epstein 2003). Such a claim may provide an opportunity to advance the dual-mind theories and overcome their main weakness, namely the multitude of dualities identified (c.f. Imbir 2016a; Keren & Schul 2009).

## Method

### Participants

The experiment involved 60 students (30 women and 30 men) from different Warsaw universities and colleges from a variety of departments (biology, technology, linguistics, economics, law etc.), aged from 19 to 27 years, with a mean of 21.48 years (*SD* = 2.12 years). All participants had normal or corrected-to-normal vision. Participation was voluntary in nature and rewarded by gift cards. Only Polish language native speakers with no knowledge of Far East languages were invited to the study. The sample size was determined in advance on the basis of earlier studies (e.g. Imbir, 2015b, 2016b) as 60 participants. The data collection was terminated when the sample size reached the quota. The experimental protocol was approved by an institutional ethical review board of Maria Grzegorzewska Academy of Special Education and study was carried out in accordance with the provisions of the World Medical Association Declaration of Helsinki.

### Linguistic Materials

The words were chosen as research materials in order to manipulate the activational properties of stimuli (arousal and subjective significance). This choice was justified by the nature of verbal materials; they all express objects and states appearing in human world (Imbir, 2015a). Words were also shown in previous studies to transmit activation (Imbir, 2015b, 2016b) even when they were presented as unrelated or in addition to the main, explicit task. Words used in current experiment were chosen from Affective Norms for Polish Words Reload dataset (ANPW-R; Imbir, 2016c). Norms were collected for a large number of items (4900 words) assessed on eight different affective scales (with use of Self-Assessment Manikin measures: Lang, 1980). Each dimension was assessed by at least 50 (25 females) students from different Warsaw universities (Appendix 1; see also: Imbir, 2015a, 2016b, 2016c). Only nouns were selected and divided into nine groups of 15 words each. In each group the valence, concreteness, length and frequency of appearance levels were closely matched for each group (there were no significant differences between groups’ items for each of controlled dimensions, c.f. Table 1). Hence, selected groups differed in levels of arousal and subjective significance so as to form a 3 (arousal levels) × 3 (subjective significance levels) factorial manipulation. The different levels of arousal and subjective significance of words were selected from stimuli rated respectively: below −1 *SD* (low intensity), from −0.5 to 0.5 *SD* (moderate intensity), and above 1 *SD* (high intensity of activation property) from the average rating in the corresponding dimension. Further, the selected words had medium ratings (between −0.5 and 0.5 *SD*) for valence and for concreteness. The selection procedure also ensured an equalization of the frequency of appearance and length (NoL) of words. Frequency estimations were based on online Polish texts (Kazojć, 2011) and represented the number of occurrences of each word in the whole database used. The distribution of values in this database was right-skewed, but was corrected by natural logarithm (LN) transformation enabling the application of parametric statistics. Thus, all analyses were conducted with use of the LN transformations of frequency estimation.

**Table 1:** Properties of the words used in the experiment. In bold, the expected main effects of arousal group for arousal ratings and effect of subjective significance groups for subjective significance ratings are presented. Lack of effects for all of the other controlled dimensions suggest validity of the material used

Dimension	Main effect of Arousal words groups	Main effect of Subjective Significance words groups	Interaction of Arousal and Subjective Significance groups
Arousal	***F*** **(2126) = 298.47,** ***p*** ** < 0.001,** ***η*** ^***2***^ **= .83**	*F*(2126) = .88, *p* = 0.4, *η* ^*2*^ = .01	*F*(4126) = .3, *p* = 0.9, *η* ^*2*^ = .009
Sub. Sign.	*F*(2126) = 3.02, *p* = .053, *η* ^*2*^ = .04	***F*** **(2126) = 263.05,** ***p*** **< .001,** ***η*** ^***2***^ **= .81**	*F*(4126) = .73, *p* = 0.6, *η* ^*2*^ = .023
Valence	*F*(2126) = 1.46, *p* = .23, *η* ^*2*^ = .02	*F*(2126) = 1.89, *p* = .16, *η* ^*2*^ = .03	*F*(4126) = .23, *p* = 0.9, *η* ^*2*^ = .007
Concreteness	*F*(2126) = .03, *p* = .97, *η* ^*2*^ < 0.001	*F*(2126) = 2.74, *p* = .07, *η* ^*2*^ = .04	*F*(4126) = .12, *p* = 0.9, *η* ^*2*^ = .004
LN frequency	*F*(2126) = 1.24, *p* = .29, *η* ^*2*^ = .02	*F*(2126) = 2.99, *p* = .054, *η* ^*2*^ = .05	*F*(4126) = .19, *p* = 0.95, *η* ^*2*^ = .006
Length	*F*(2126) = .57, *p* = .57, *η* ^*2*^ = .01	*F*(2126) = 1.29, *p* = .28, *η* ^*2*^ = .02	*F*(4126) = .19, *p* = 0.94, *η* ^*2*^ = .006

To ensure correct construction of the manipulation, 3 (arousal levels) × 3 (subjective significance levels) ANOVA analyses were computed for each dimension measured. Table 1 presents the pattern of obtained results. Table 2 presents the mean values (*M*) and standard deviations (*SD*) of assessments for each manipulated group of words.

**Table 2:** Descriptive statistics (M, SD) for groups of words used in factorial manipulation

Subjective significance Category	Dimension of assessments	Arousal category							
		Low		Medium		High		Total	
		*M*	*(SD)*	*M*	*(SD)*	*M*	*(SD)*	*M*	*(SD)*
Low	Arousal	3.20	.29	3.84	.27	4.79	.53	3.94	.76
	Sub. Sign.	2.87	.33	2.96	.18	2.93	.60	2.92	.40
	Valence	5.25	.48	5.11	.44	5.01	.63	5.12	.52
	Concreteness	4.07	1.02	3.88	.73	3.91	.90	3.95	.87
	LN frequency	6.04	1.86	6.32	1.41	5.95	1.36	6.10	1.53
	Length	5.73	2.09	6.20	1.32	6.07	2.02	6.00	1.81
Medium	Arousal	3.20	.16	3.85	.26	4.85	.35	3.97	.73
	Sub. Sign.	3.56	.32	3.71	.22	3.74	.34	3.67	.30
	Valence	5.42	.47	5.38	.62	5.10	.66	5.30	.59
	Concreteness	3.92	.90	3.94	.90	3.98	.75	3.95	.83
	LN frequency	6.50	1.52	6.29	1.58	5.68	1.91	6.16	1.68
	Length	6.27	2.09	6.13	1.68	6.87	2.45	6.42	2.07
High	Arousal	3.27	.27	3.85	.32	4.97	.33	4.03	.78
	Sub. Sign.	4.55	.31	4.64	.40	4.88	.44	4.69	.41
	Valence	5.38	.36	5.41	.35	5.31	1.11	5.37	.69
	Concreteness	4.28	.75	4.33	1.00	4.37	.98	4.33	.89
	LN frequency	6.99	2.02	7.08	1.23	6.55	1.90	6.87	1.73
	Length	6.47	2.13	6.67	1.95	6.87	2.00	6.67	1.99
Total	Arousal	3.22	.24	3.85	.28	4.87	.41	3.98	.75
	Sub. Sign.	3.66	.77	3.77	.75	3.85	.93	3.76	.82
	Valence	5.35	.44	5.30	.49	5.14	.82	5.26	.61
	Concreteness	4.09	.89	4.05	.88	4.09	.89	4.08	.88
	LN frequency	6.51	1.81	6.56	1.43	6.06	1.74	6.38	1.67
	Length	6.16	2.08	6.33	1.65	6.60	2.15	6.36	1.96

As one can see, the intended differences were found for arousal dimension in groups differing in arousal levels and subjective significance dimension in groups differing in subjective significance levels. There was no statistically significant difference between groups for valence, concreteness, LN of frequency and length. The same list of words was used in an earlier study concerning effects of arousal and subjective significance on cognitive control in a modified Stroop task (Imbir, 2016b).

### Warmth Vs. Competence Ambiguous Task

**Fig. 2:**
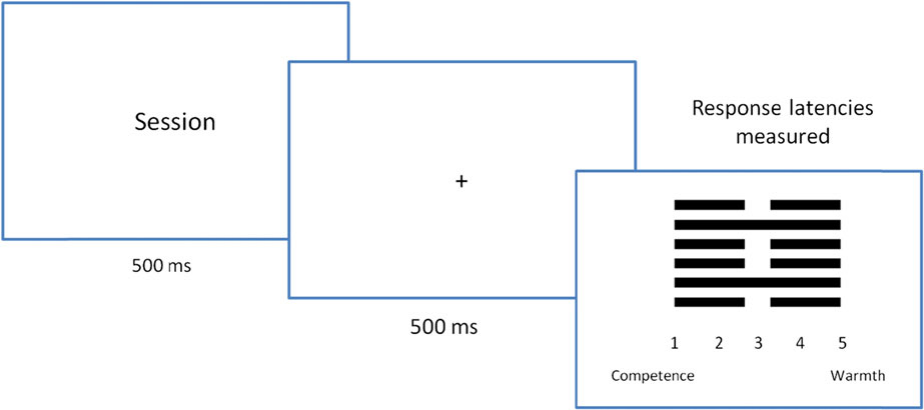
The single trial of ambiguous task comprised (1) presentation of word charged with activation, (2) delay time for storing the word in memory and (3) hexagram sign representing a personality trait rated on a competence vs. warmth scale

To assess the impact of words’ activation level on social perception in warmth vs. competence dimensions, an ambiguous task was designed as a modification of a paradigm proposed by Błaszczak and Imbir (2012). The idea is simple and originates from affective priming paradigm (Murphy & Zajonc, 1993). A sign unknown to the participants in the form of a hexagram (see Fig. 2) was introduced as a pictorial representation of human character or personality trait. The task was to guess by intuition the meaning of the presented sign and rate it on a 5 point Likert scale described as varying from a competence (1) to warmth (5) related term. The order of scale ends presentation was always the same. Competence was present at the beginning in order to minimize the effect of prioritization of warmth processing found in earlier studies. Although warmth and competence are thought to be rather distinct dimensions (c.f. Wojciszke, 2005), for the purpose of this study they were integrated into a single scale. At the exploratory level of analysis, the main focus of participants’ interpretation was measured. In other words only the dominating perspective at a given time could have been measured in this study. Both categories meanings were explained, and the definitions were provided, thus participants knew what this scale represented. Competence was defined as describing skills and abilities. Features related to competence include people solving problems better, achieving better results. People who are competent were described as smarter than others and able to rule over others. Warmth was defined as the preference for relationships. Features related to warmth were claimed to make people better in interacting with others or on a team. People that are warm were described as having better relationships, being more popular and attracting other people to them. Reaction latencies and types of answers were measured in a hexagram meaning guessing task.

### Design and Apparatus

A 3 (arousal levels) × 3 (subjective significance levels) within-subject design was used. The dependent variables were (a) type of answer in hexagram interpretation task and (b) time spent on hexagram interpretation on competence – warmth scale. The whole experiment was fully randomized, thus words from different categories and word-hexagram pairs were displayed to the participants in a random fashion. A standard 15 in. laptop computer was used in order to present the experimental procedure. The session was prepared with use of E-Prime 2.0 software.

### Procedure

Participants were invited into the laboratory for individual sessions lasting about 35 min. They were told that the study focuses on the boundaries of human intuition seeking an answer to whether the meaning of Far East culture signs can be guessed despite the memory load. For that reason, the second task was introduced as a brief (500 ms) presentation of words. The additional task was to try to remember as much as possible all words that were presented during the experiment. For remembering, there was an additional 500 ms after word presentation end in each trial.

Participants were comfortably seated in front of the computer screen and then the experiment started. Participants were instructed to place fingers of both hands over the marked response keys (1, 2, 3, 4 and 5) on a keyboard of a laptop computer. The whole experiment was based on ambiguous task procedure. Participants were encouraged to try to guess meaning of signs originating from far-east cultures. No instruction was provided concerning speed of processing. The single trial of this task entailed three events: (1) presentation of word stimuli lasting 500 ms, (2) fixation point lasting 500 ms and (3) hexagram sign presentation with accompanying answering scale. The words were introduced as an additional task designed to create the working memory load. Words were displayed in a fully random order to avoid sequence effects. Figure 2 presents a single trial of the ambiguous task used in the current experiment.

The whole experiment had 270 trials of the ambiguous task placed in two different blocks of 135 trials presented in random order. One of these blocks is discussed in the current manuscript. Another contained different lists of words focusing on valence and origin of affective state differences and will be discussed in another paper. Two blocks differed as well as the type of ambiguous stimulus used, in order not to give participants the chance to habituate to hexagrams. In the valence block, the Japanese letter compounds were presented. Was All 145 words in each block were presented in a random order. Also ambiguous stimuli were displayed in a random order. No effects of interference between both blocks were found. After each session, the direct associations of words with warmth-competence scale were assessed for each participant with use of scale from main part of experiment. Appendix 1 presents values of direct associations for each word.

### Data Treatment and Analytical Strategy

A total of 8100 trials from all 60 subjects were included in the initial analysis. The first step was to inspect reaction latencies. The initial mean reaction latencies was *M* = 2332 ms (*SD* = 2963 ms), ranging from 8 ms to 70,662 ms. Data were normalized and outliers, reaction latencies lower than 350 ms (691 trials, set as a reasonable time needed to respond consciously, Z < −1.42) or longer than 13,900 ms (87 trials, Z > 2.3 was set as criterion), were excluded from further consideration. Relatively weak restriction of long reaction latency outliers was based on the assumption that decision making in an ambiguous task is rather difficult, thus requiring time. Reaction time for remaining trials were transformed by *ln*, then all data (responses, reaction latencies and log transformed reaction latencies) were aggregated across participants and conditions. LN transformation is a standard procedure for reaction time data to analyze a right-skewed (c.f., Heathcote, Popiel, & Mewhort, 1991) distribution using parametric statistics.

Data were analyzed with a 3 (word arousal levels) × 3 (word subjective significance levels) within-subjects ANOVA with repeated measures. The dependent variables were type of answer chosen and log transformed reaction latency, both measured in the warmth-competence hexagram meaning guessing ambiguous task.

## Results

### Type of Answer in Hexagram Meaning Guessing

**Fig. 3:**
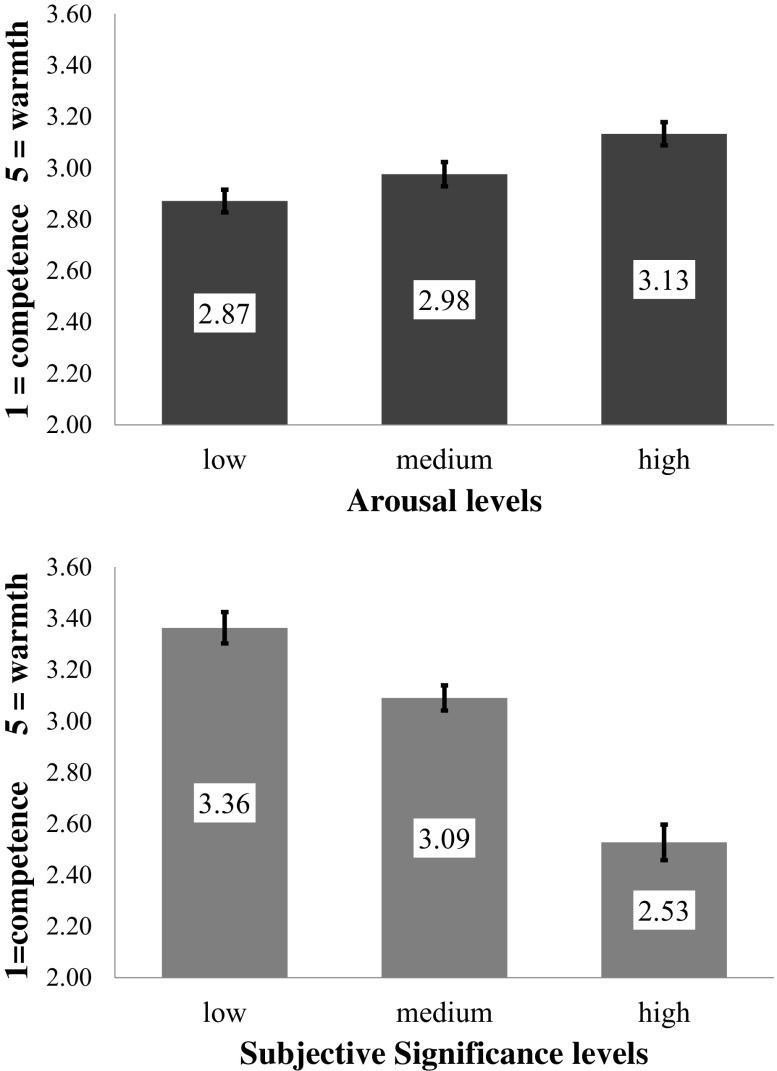
Mean responses in competence (1) vs. warmth (5) dimensions in conditions differing in arousal and subjective significance levels of words. Error bars represents SEM

Results showed a statistically significant main effect of arousal level for responses in warmth-competence dimension: *F*(2,58) = 13.44, *p* = .001, *η* ^*2*^ = 0.32. Simple contrast analysis with Bonferroni correction for multiple comparison showed that mean responses differed between all level of arousal: low arousing words condition (*M* = 2.87, *SEM* = .044) and medium arousing words condition (*M* = 2.98, *SEM* = .047): *F*(1,59) = 8.35, *p* = .016, *η* ^*2*^ = 0.12; low arousing and high arousing words (*M* = 3.13, *SEM* = .045): *F*(1,59) = 27.29, *p* = .001, *η* ^*2*^ = 0.32 and between medium and high arousing words: *F*(1,59) = 22.99, *p* = .002, *η* ^*2*^ = 0.28. Also the main effect of the subjective significance factor was statistically significant: *F*(2,58) = 35.31, *p* = .001, *η* ^*2*^ = 0.55. Simple contrast analysis with Bonferroni correction for multiple comparison showed that mean responses differed between all levels of subjective significance: low subjective significant words condition (*M* = 3.36, *SEM* = .061) and medium subjective significant words condition (*M* = 3.09, *SEM* = .049): *F*(1,59) = 21.63, *p* = .001, *η* ^*2*^ = 0.27; low and high subjective significant words (*M* = 2.53, *SEM* = .069): *F*(1,59) = 71.8, *p* = .001, *η* ^*2*^ = 0.55 and between medium and high subjective significant words: *F*(1,59) = 67.03, *p* = .001, *η* ^*2*^ = 0.53. No statistically significant interaction between arousal and subjective significance of words used was found: *F*(4,56) = 2.06, *p* = .1, *η* ^*2*^ = 0.13. The pattern of results is shown in Fig. 3.

### Reaction Latencies in Hexagram Meaning Guessing

**Fig. 4:**
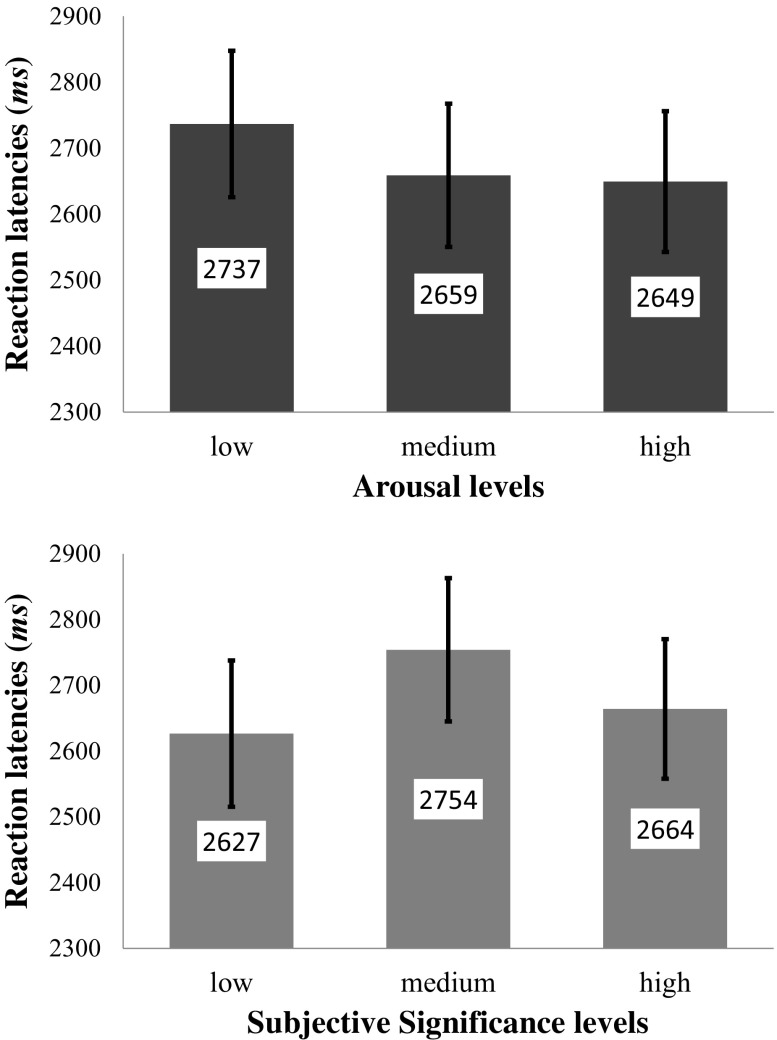
Mean response latencies (ms) in ambiguous competence-warmth hexagram matching task for conditions differing in arousal and subjective significance levels of words. Error bars represents SEM

In order to make the presentation of results clearer, results in the text are presented in log transformation, but Fig. 4 shows data in milliseconds (*ms*). ANOVA analysis for reaction latencies showed a statistically significant main effect of arousal level for responses in warmth-competence dimension: *F*(2,58) = 3.53, *p* = .036, *η* ^*2*^ = 0.11. Simple contrast analysis with Bonferroni correction for multiple comparison showed that mean response latencies did not differ between: low arousing words (log transformed data: *M* = 7.56, *SEM* = .074) and medium arousing words (*M* = 7.53, *SEM* = .075): *F*(1,59) = 4.14, *p* = .33, *η* ^*2*^ = 0.042 and between medium and high arousing words (*M* = 7.51, *SEM* = .074): *F*(1,59) = 2.61, *p* = .9, *η* ^*2*^ = 0.066. The difference between low arousing and high arousing words was statistically significant: *F*(1,59) = 6.93, *p* = .032, *η* ^*2*^ = 0.11. The main effect of the subjective significance factor was also statistically significant: *F*(2,58) = 6.72, *p* = .002, *η* ^*2*^ = 0.19. Simple contrast analysis with Bonferroni correction for multiple comparison showed that mean response latencies differed between: low subjective significant words (*M* = 7.51, *SEM* = .075) and medium subjective significant words (*M* = 7.58, *SEM* = .072): *F*(1,59) = 12.89, *p* = .002, *η* ^*2*^ = 0.18. No statistically significant differences were found between medium vs. high (*M* = 7.52, *SEM* = .075) subjective significant words: *F*(1,59) = 1.38, *p* = .26, *η* ^*2*^ = 0.023 and low vs. high subjective significant words: *F*(1,59) = .64, *p* = .9, *η* ^*2*^ = 0.01. No statistically significant interaction between arousal and subjective significance of words used was found: *F*(4,56) = 1.6, *p* = .19, *η* ^*2*^ = 0.1. The pattern of results is shown in Fig. 4.

### Direct Associations between Words Meaning and Warmth-Competence

In order to check the alternative explanation of results, direct associations were analyzed with use of ANOVA. Assessments were provided by each participant after main experiment for each of word used. Results showed a no statistically significant main effect of arousal level: *F*(2126) = 0.52, *p* = .6, *η* ^*2*^ = 0.008, nor subjective significance level: *F*(2126) = 1.28, *p* = .28, *η* ^*2*^ = 0.02 for direct associations in warmth-competence. Also no statistically significant interaction between arousal and subjective significance of words used was found: *F*(4126) = .36, *p* = .8, *η* ^*2*^ = 0.011. Mean association was 2.82 with *SD* = .56.

## Discussion

This study showed that perception of ambiguous objects is susceptible to the activation included in words presented before the task as an unrelated distractor. Arousal as an activational mechanism of experiential mental processing was expected to promote perception of hexagrams in terms of warmth, while the opposite effect was expected for subjective significance, which is claimed to be an activational mechanism for rational mental processing, and which promoted perception of competence in ambiguous objects. Results confirmed these expectations. The more arousing the stimulus was, the more hexagrams were perceived as representing a warmth related trait. A very similar, but opposite linear tendency could be seen for subjective significance: the more subjectively significant words were, the more hexagrams were perceived as related to competence. This pattern of results supports the claim that the experiential mind (Epstein, 2003) and arousal, as its activation mechanism, are connected with warmth in social perception (Fiske et al., 2007), while the rational mind and its activation mechanism of subjective significance (Imbir, 2015a, 2016a) are associated with competence in social perception. It is worth highlighting that no direct associations for words used in the experiment were found, thus, we may exclude semantic priming as a source of results (c.f. Payne et al., 2005).

Reaction latencies data showed that, in high arousing words conditions, participants made their decision more quickly in comparison to low arousing words. This effect can be explained in two ways. Firstly, arousal was found to promote warmth perception. Since warmth processing was found to be prioritized (Willis & Todorov, 2006; Ybarra et al., 2001), one may expect such an effect. Secondly, the arousal level of words used could be the optimal one, facilitating processing. It is worth noticing that, due to the main aim to create a factorial design for arousal and subjective significance manipulation with precise control for valence and concreteness, the relative level of arousal in words used was, in fact, moderate (4.87 in 9 point Likert scale, c.f. Imbir, 2015a, 2016c). This could account for the shortening of the reaction latencies observed here. Moreover, trials after low subjective significance words were processed quicker in comparison to those after medium subjective significant stimuli. One may predict that, since increasing levels of subjective significance made the responses more competence related, the use of more rational mental processing mechanisms resulting in longer time needed to give the answer. From this point of view, the results are understandable. Surprisingly, no such effect was found for high subjective significance words. This is probably due to the fact that the nature of the task used is not quite relevant to the rational mind; there is no algorithmic strategy to answer the question concerning hexagram meaning.

Earlier studies on word recognition (c.f. Kuperman, Estes, Brysbaert, & Warriner, 2014) showed that increasing arousal level cause the slowdown in word processing, namely in word recognition latencies. Such results are congruent with studies concerning effects of arousal on reaction latencies in emotional Stroop test (c.f. Burt, 2002; Imbir, 2016b). In current experiment the time spent on word stimuli processing was not measured, although results of another studies involving incidental affect elicitation by verbal stimuli of matched concreteness and frequency of appearance (c.f. Antosz & Imbir, 2016) showed no differences in reading times, but influence of incidental affect on subsequent ambiguous task. The theoretical explanation for reversed effects of arousal for reaction latencies in paradigms involving processing of arousing words themselves (e.g. Imbir, 2016b; Kuperman et al., 2014) and those focused on a consequences of arousal on a subsequent task (c.f. incidental activation evoked in current study) may be that in first case increasing arousal level may cause increase in a reaction latencies due to paying more attention to arousing stimuli (such objects may be related to life threats: c.f. Imbir, 2016a), while in the second case activation caused by arousal may facilitate the processing characteristic to experiential mind (Epstein, 2003; Imbir, 2016a).

Results of the study shows a great importance for understanding the mental mechanisms that support the dual-mind perspective. The most important critique levelled at dual-mind theories concerns the multitude of dualities postulated by different researchers (c.f. Keren & Schul 2009) working in different fields. This study aimed at searching for links between dualities of different processes (c.f. Imbir, 2016a), in order to validate the expectation that all of them are related to the more general duality of experiential and rational mind postulated by Epstein (2003). It seems that arousal and subjective significance are distinct activation mechanisms that can trigger the perception of ambiguous stimuli in terms of warmth and competence, respectively, although no direct associations for stimuli meaning were found in these two dimensions of social perception. This opens a new field of research and suggests the need for change to the most popular approach in dual-mind theories. Namely, each existing dichotomy should be validated in the context of its ability to evoke mind-system-specific processing in different aspect of functioning. Such an approach was recently postulated in a new model discussing the relations between emotional and cognitive processes from the dual-mind perspective (Imbir, 2016a). Results of the current study lead us to the conclusion that all of the dichotomies listed in introduction should be treated as derivates of more general mind systems, rather than as distinct concepts (Epstein, 2003; Imbir, 2016a; Kahneman, 2011).

The results of this study are also important for understanding the perception of other people, within the field of social psychology. First of all, warmth and competence dimensions have not yet been discussed as emanations of dual-mind processes, therefore, further hypotheses for testing this idea in experimental studies may be derived from the results of the current study that supports this claim. In particular, when warmth is related to the experiential mind, while competence is related to the rational, then we should be able to observe not only the prioritization of warmth assessments, but also other characteristics of experiential mind processing, such as the domination of associative thinking mechanisms in warmth assessments (compared to cause and effects mechanisms in the case of competence assessments) or lower ability to change initial warmth assessments in comparison to easier changes (perhaps labiality based on upcoming evidences) in competence judgments (Epstein, 2003). All of the relationships mentioned above are only hypothetical, however, they seems to be worthy of further testing. Second of all, results of this study may shed new light on the mechanisms of stereotype creation or acquisition. To put it simply, changes of incidental activation (Russell, 2003) may not be consciously perceived by individuals, but they may influence the interpretation of unfamiliar objects, including members of other social groups, therefore, some instances for understanding the development of stereotypes may be derived from the results of this study.

Limitations of the current study includes the operationalization of warmth and competence in a single scale, instead of two distinct dimensions. In fact, this only allowed for concluding that a general tendency in social perception occurs after contact with activation charged words, but nothing could be said about the dynamic of perception in both dimensions. It is possible that the domination of a single dimension can be observed when the second dimension is overwhelmed, but still active, when judging. This limitation should be addressed in subsequent studies. Furthermore, it is worth searching for the relations postulated in previous paragraph among fundamental dimensions of social cognition, derived from experiential and rational mind models (Epstein, 2003). Lastly, an important issue worth investigating is the role of valence, which often accompanies activation (c.f. core affect concept: Russell, 2003), and that may influence the relationship described above.

In conclusion, it is worth highlighting that the current study demonstrated the susceptibility of social perception to incidental activation. Reading and remembering words charged with activation influenced the perception of ambiguous stimuli. The same effect should be present in other domains of social perception. Everyday experience is full of occasions when incidental affect can influence our current state (Ferguson et al., 2005; Russell, 2003), thus, our judgments towards other people or objects may be biased by the nature of activation accompanying the situation. Another important aspect of this experiment concerns the successful attempt to operationalize rational mind activation (Imbir, 2015a), which can be transmitted and alter the subsequent judgments of unknown objects.

## Electronic supplementary material

ESM 1 (XLSX 20 kb)
